# Low-temperature redetermination of hexa­kis(μ-chloro­acetato-κ^2^ *O*:*O*′)-μ_3_-oxido-tris[aquachromium(III)] nitrate 3.5-hydrate

**DOI:** 10.1107/S1600536808023805

**Published:** 2008-07-31

**Authors:** Seik Weng Ng

**Affiliations:** a Department of Chemistry, University of Malaya, 50603 Kuala Lumpur, Malaysia

## Abstract

A low-temperature redetermination of the trinuclear cluster compound described as [Cr_3_(C_2_H_2_ClO_2_)_6_O(H_2_O)_3_]NO_3_·3H_2_O [Glowiak, Kubiak & Jezowska-Trzebiatowska (1977 ▶). *Bull. Acad. Pol. Sci. Ser. Sci. Chim.* **25**, 359–371] shows that the salt is a 3.5-hydrate, [Cr_3_(C_2_H_2_ClO_2_)_6_O(H_2_O)_3_]NO_3_·3.5H_2_O. The trinuclear cluster cation is disordered in four of the six monochloro­acetate groups. One is disordered over two positions in respect of the chloro­methyl atoms (occupancy ratio 0.50:0.50); another is disordered over three positions in respect of the chloro­methyl atoms (occupancy ratio 0.50:0.37:0.13) whereas two are disordered over two positions in respect of the Cl atoms only (occupancy ratios 0.84:0.16 and 0.60:0.40). Of the four independent uncoordinated water mol­ecules, one has an occupancy factor of 0.5. The trinuclear cation has an oxido O atom that is connected to three water-coordinated Cr^III^ atoms, the three metal atoms forming the points of an equilateral triangle. Six carboxyl­ate groups each chelate a Cr—O—Cr fragment. The cations, anions and uncoordinated water mol­ecules are linked by hydrogen bonds.

## Related literature

For the room-temperature study, see: Glowiak *et al.* (1977 ▶).
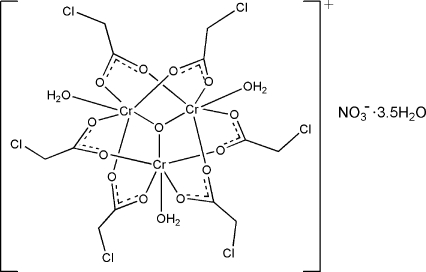


## Experimental

### 

#### Crystal data

[Cr_3_(C_2_H_2_ClO_2_)_6_O(H_2_O)_3_]NO_3_·3.5H_2_O
*M* *_r_* = 912.03
Monoclinic, 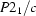

*a* = 12.4938 (2) Å
*b* = 14.7622 (2) Å
*c* = 17.2687 (3) Å
β = 96.293 (1)°
*V* = 3165.78 (9) Å^3^
*Z* = 4
Mo *K*α radiation
μ = 1.61 mm^−1^
*T* = 100 (2) K
0.12 × 0.06 × 0.03 mm

#### Data collection

Bruker SMART APEX CCD area-detector diffractometer
Absorption correction: multi-scan (*SADABS*; Sheldrick, 1996 ▶) *T*_min_ = 0.820, *T*_max_ = 0.953
39914 measured reflections
7271 independent reflections
5349 reflections with *I* > 2σ(*I*)
*R*_int_ = 0.053

#### Refinement

*R*[*F* ^2^ > 2σ(*F* ^2^)] = 0.040
*wR*(*F* ^2^) = 0.112
*S* = 1.03
7271 reflections
500 parameters
119 restraints
H atoms treated by a mixture of independent and constrained refinement
Δρ_max_ = 0.99 e Å^−3^
Δρ_min_ = −0.34 e Å^−3^

### 

Data collection: *APEX2* (Bruker, 2007 ▶); cell refinement: *SAINT* (Bruker, 2007 ▶); data reduction: *SAINT*; program(s) used to solve structure: *SHELXS97* (Sheldrick, 2008 ▶); program(s) used to refine structure: *SHELXL97* (Sheldrick, 2008 ▶); molecular graphics: *X-SEED* (Barbour, 2001 ▶); software used to prepare material for publication: *publCIF* (Westrip, 2008 ▶).

## Supplementary Material

Crystal structure: contains datablocks global, I. DOI: 10.1107/S1600536808023805/hy2147sup1.cif

Structure factors: contains datablocks I. DOI: 10.1107/S1600536808023805/hy2147Isup2.hkl

Additional supplementary materials:  crystallographic information; 3D view; checkCIF report

## Figures and Tables

**Table 1:** Hydrogen-bond geometry (Å, °)

*D*—H⋯*A*	*D*—H	H⋯*A*	*D*⋯*A*	*D*—H⋯*A*
O1*w*—H11⋯O5*w*^i^	0.84 (4)	1.83 (2)	2.616 (6)	157 (5)
O1*w*—H12⋯O6*w*^ii^	0.84 (3)	1.99 (3)	2.816 (4)	169 (5)
O2*w*—H21⋯O15^iii^	0.84 (3)	1.90 (3)	2.732 (4)	170 (4)
O2*w*—H22⋯Cl5′^iv^	0.84 (3)	2.75 (3)	3.295 (6)	124 (6)
O3*w*—H31⋯O4*w*	0.844 (14)	1.84 (2)	2.669 (4)	168 (5)
O3*w*—H32⋯O6*w*	0.84 (3)	1.88 (3)	2.711 (4)	171 (4)
O4*w*—H41⋯O7^v^	0.85 (5)	2.20 (5)	3.046 (4)	175 (5)
O4*w*—H42⋯O16^ii^	0.83 (4)	2.27 (4)	2.976 (5)	142 (6)
O5*w*—H51⋯O7*w*	0.85 (4)	2.06 (7)	2.79 (1)	144 (11)
O5*w*—H52⋯O12	0.85 (7)	2.47 (7)	3.27 (1)	156 (10)
O6*w*—H61⋯O14	0.83 (4)	2.31 (2)	3.093 (6)	158 (5)
O6*w*—H62⋯O7*w*	0.85 (4)	2.15 (3)	2.86 (1)	141 (4)

Symmetry codes: (i) 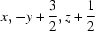; (ii) 
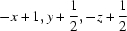
; (iii) 
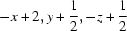
; (iv) 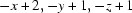; (v) 
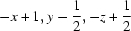
.

**Fig. 1.**
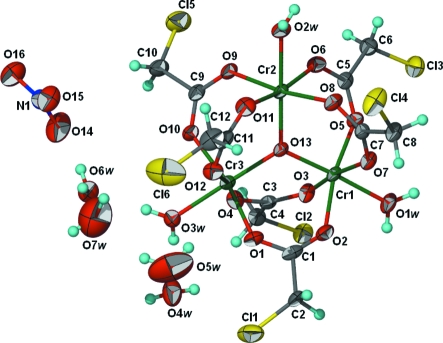
Molecular structure of the title compound. Displacement ellipsoids are drawn at the 50% probability level. The minor disorder components are not shown. The water molecule O7w has 0.5 occupancy.

## supplementary crystallographic information

### Comment

The low-temperature redetermination of the trinuclear cluster compound described as [Cr_3_O(C_2_H_2_ClO_2_)_6_(H_2_O)_3_](NO_3_).3H_2_O by Glowiak *et al.* (1977) shows that the salt is a 3.5-hydrate, [Cr_3_O(C_2_H_2_ClO_2_)_6_(H_2_O)_3_](NO_3_).3.5H_2_O (Scheme1; Fig. 1). The trinuclear cluster cation is disordered in four of the six monochloroacetate groups. One is disordered over two positions in respect of the chloromethyl atoms (occupancy ratio 0.50:0.50); another is disordered over three positions in respect of the chloromethyl atoms (occupancy ratio 0.50:0.37:0.13) whereas two are disordered over two positions in respect of the Cl atoms only (occupancy ratios 0.84:0.16 and 0.60:0.40). Of the four independent lattice water molecules, one has an occupancy factor of 0.50. The trinuclear cation has an oxido O atom that is connected to three water-coordinated Cr^III^ atoms, the three metal atoms forming the points of an equilateral triangle. Six carboxylate groups each chelates a Cr–O–Cr fragment. The cations, anions and lattice water molecules are linked by hydrogen bonds (Table 1).

### Experimental

Crystals of the title compound obtained by the method of Glowiak *et al.* (1977) were supplied by Dr Rosiyah Yahya.

### Refinement

The trinuclear cluster cation is disordered in four of the six monochloroacetate groups. Two are disordered over two positions in respect of the Cl atom (Cl1, Cl1') and attached C atom (C2, C2'). The C—C distances were restrained to within 0.01±0.01 Å of each other, as were the C—Cl distances. The temperature factors of the primed C atom were restrained to be equal to those of the umprimed C atom. The disorder was refined to nearly 0.50:0.50.

The other monochloroacetate group is disordered over three positions in respect of the Cl atom (Cl6, Cl6', Cl6") and attached C atom (C12, C12', C12"). The C—C distances were restrained to within 0.01±0.01 Å of each other, and this restraint was applied to the three C—Cl distances. The temperature factors of the C atoms were restrained to be identical. The disorder was refined to approximately 0.50:0.33:0.17.

Meanwhile, the other two monochloroacetate groups are disordered over two positions but for the Cl atoms only (Cl4, Cl4' and Cl5, Cl5'). For each, the C—Cl distances were restrained to within 0.01 Å of each other.

In the later stages of the refinement, the difference Fourier map had an electron density at about 2.5 Å from Cl1 and Cl6, and at about 2.8 Å from O5w and O6w. The electron density was satisfactorily modeled as half a water molecule (O7w). Since the occupancies of Cl1 and Cl6 were refined to nearly 0.5, the occupancies of O7w, Cl1 and Cl6 should, in fact, be exactly 0.5. The O7w atom should be within hydrogen bonding range of O5w and O6w, but should not be near Cl1 and Cl6. As the occupancy of Cl6 was fixed as 0.5, the occupancies of the other Cl6' and Cl6" components were then allowed to refine, subject to a total of 0.5. The ratio was refined to 0.365 (3):0.135 (3).

The anisotropic temperature factors of the three-and-a-half lattice water molecules were restrained to be nearly isotropic. For the six full-occupied water molecules, their H atoms were located in a difference Fourier map and refined with distance restraints of O—H = 0.84 (1) Å and H···H = 1.37 (1) Å. Their temperature factors were tied to those of the parent atoms by a factor of 1.5. As the half-occupied water molecule (O7w) is an acceptor to two hydrogen bond donors, its H atoms could be placed in chemically sensible positions; they were not refined. The O7w does not form hydrogen bonds to donor atoms. H atoms on C atoms were positioned geometrically and refined as riding atoms, with C—H = 0.99 Å and *U*_iso_(H) = 1.2*U*_eq_(C). The final difference Fourier map was essentially featureless, with no peak larger than 1 e Å^-3^ and no hole deeper than -1 e Å^-3^.

### Crystal data

**Table:**

[Cr_3_(C_2_H_2_ClO_2_)_6_O(H_2_O)_3_]NO_3_·3.5H_2_O	*F*_000_ = 1832
*M**_r_* = 912.03	*D*_x_ = 1.914 Mg m^−^^3^
Monoclinic, *P*2_1_/*c*	Mo *K*α radiation λ = 0.71073 Å
Hall symbol: -P 2ybc	Cell parameters from 7530 reflections
*a* = 12.4938 (2) Å	θ = 2.4–25.8º
*b* = 14.7622 (2) Å	µ = 1.61 mm^−^^1^
*c* = 17.2687 (3) Å	*T* = 100 (2) K
β = 96.293 (1)º	Irregular chip, green
*V* = 3165.78 (9) Å^3^	0.12 × 0.06 × 0.03 mm
*Z* = 4	

### Data collection

**Table:**

Bruker SMART APEX CCD area-detector diffractometer	5349 reflections with *I* > 2σ(*I*)
Radiation source: fine-focus sealed tube	*R*_int_ = 0.053
Monochromator: graphite	θ_max_ = 27.5º
φ and ω scans	θ_min_ = 1.8º
Absorption correction: multi-scan(SADABS; Sheldrick, 1996)	*h* = −16→16
*T*_min_ = 0.820, *T*_max_ = 0.953	*k* = −19→19
39914 measured reflections	*l* = −21→22
7271 independent reflections	

### Refinement

**Table:**

Refinement on *F*^2^	Secondary atom site location: difference Fourier map
Least-squares matrix: full	Hydrogen site location: inferred from neighbouring sites
*R*[*F*^2^ > 2σ(*F*^2^)] = 0.040	H atoms treated by a mixture of independent and constrained refinement
*wR*(*F*^2^) = 0.112	*w* = 1/[σ^2^(*F*_o_^2^) + (0.0523*P*)^2^ + 3.1321*P*] where *P* = (*F*_o_^2^ + 2*F*_c_^2^)/3
*S* = 1.03	(Δ/σ)_max_ = 0.001
7271 reflections	Δρ_max_ = 0.99 e Å^−^^3^
500 parameters	Δρ_min_ = −0.34 e Å^−^^3^
119 restraints	Extinction correction: none
Primary atom site location: structure-invariant direct methods	

### Fractional atomic coordinates and isotropic or equivalent isotropic displacement parameters (Å^2^)

**Table:**

	*x*	*y*	*z*	*U*_iso_*/*U*_eq_	Occ. (<1)
Cr1	0.65855 (4)	0.69687 (3)	0.39526 (3)	0.02667 (13)	
Cr2	0.88330 (4)	0.58714 (3)	0.37503 (3)	0.02515 (13)	
Cr3	0.66063 (4)	0.53305 (3)	0.26482 (3)	0.02583 (13)	
Cl1	0.4146 (3)	0.7073 (3)	0.09142 (17)	0.0663 (8)	0.50
Cl1'	0.4626 (3)	0.7531 (2)	0.09294 (17)	0.0689 (8)	0.50
Cl2	0.33828 (7)	0.56139 (6)	0.47320 (6)	0.0418 (2)	
Cl3	0.77180 (9)	0.70060 (7)	0.66950 (6)	0.0518 (3)	
Cl4	1.0378 (4)	0.86104 (15)	0.3111 (5)	0.0628 (13)	0.836 (19)
Cl5	0.9066 (6)	0.2693 (5)	0.4493 (4)	0.0461 (19)	0.60 (6)
Cl4'	1.0551 (6)	0.8685 (10)	0.352 (2)	0.057 (4)	0.164 (19)
Cl5'	0.882 (3)	0.2778 (14)	0.4609 (12)	0.076 (4)	0.40 (6)
Cl6	0.8796 (3)	0.5426 (4)	0.0487 (2)	0.1104 (14)	0.50
Cl6'	0.8604 (3)	0.6140 (3)	0.0492 (2)	0.0697 (12)	0.365 (3)
Cl6"	1.0377 (7)	0.6016 (6)	0.1326 (6)	0.059 (3)	0.135 (3)
O1	0.5781 (2)	0.63301 (17)	0.21039 (15)	0.0379 (6)	
O2	0.5744 (2)	0.74057 (16)	0.30038 (17)	0.0423 (6)	
O3	0.54075 (18)	0.61446 (15)	0.41606 (15)	0.0340 (6)	
O4	0.54119 (19)	0.50527 (16)	0.32604 (15)	0.0345 (6)	
O5	0.72939 (19)	0.67414 (15)	0.50140 (15)	0.0339 (5)	
O6	0.87725 (18)	0.59321 (17)	0.48763 (15)	0.0351 (6)	
O7	0.76364 (18)	0.79443 (14)	0.37780 (15)	0.0331 (6)	
O8	0.91622 (18)	0.71719 (15)	0.37064 (16)	0.0370 (6)	
O9	0.87243 (19)	0.45427 (15)	0.38598 (16)	0.0362 (6)	
O10	0.7303 (2)	0.41852 (15)	0.30173 (15)	0.0357 (6)	
O11	0.9199 (2)	0.57897 (18)	0.26822 (16)	0.0411 (6)	
O12	0.7686 (2)	0.55303 (18)	0.19041 (15)	0.0381 (6)	
O13	0.73395 (16)	0.60570 (13)	0.34506 (13)	0.0254 (5)	
O14	0.6731 (3)	0.1472 (3)	0.2318 (3)	0.0833 (13)	
O15	0.8166 (3)	0.1658 (2)	0.1787 (2)	0.0597 (9)	
O16	0.7875 (3)	0.0391 (2)	0.2332 (2)	0.0697 (10)	
O1w	0.5730 (2)	0.79263 (18)	0.4438 (2)	0.0475 (7)	
H11	0.592 (3)	0.829 (3)	0.480 (2)	0.071*	
H12	0.5059 (10)	0.795 (3)	0.433 (3)	0.071*	
O2w	1.04519 (18)	0.57105 (16)	0.40302 (16)	0.0341 (6)	
H21	1.092 (2)	0.595 (3)	0.3781 (17)	0.051*	
H22	1.069 (3)	0.570 (3)	0.4506 (7)	0.051*	
O3w	0.5804 (2)	0.45767 (17)	0.18032 (16)	0.0387 (6)	
H31	0.5132 (10)	0.459 (3)	0.182 (3)	0.058*	
H32	0.598 (3)	0.4075 (16)	0.163 (3)	0.058*	
O4w	0.3657 (3)	0.4663 (2)	0.1620 (2)	0.0705 (10)	
H41	0.328 (4)	0.419 (3)	0.154 (3)	0.106*	
H42	0.352 (5)	0.489 (4)	0.204 (2)	0.106*	
O5w	0.6040 (6)	0.5620 (5)	0.0300 (4)	0.141 (2)	
H51	0.613 (9)	0.506 (2)	0.021 (6)	0.211*	
H52	0.635 (9)	0.574 (7)	0.075 (3)	0.211*	
O6w	0.6466 (3)	0.3047 (2)	0.1130 (2)	0.0585 (8)	
H61	0.672 (4)	0.265 (3)	0.144 (2)	0.088*	
H62	0.691 (3)	0.316 (3)	0.080 (2)	0.088*	
O7w	0.7204 (12)	0.4105 (8)	−0.0095 (8)	0.157 (5)	0.50
H71	0.7843	0.4292	−0.0046	0.235*	0.50
H72	0.7038	0.3898	−0.0545	0.235*	0.50
N1	0.7603 (2)	0.1175 (2)	0.2160 (2)	0.0417 (8)	
C1	0.5514 (3)	0.7089 (2)	0.2344 (2)	0.0349 (8)	
C2	0.4863 (12)	0.7624 (7)	0.1699 (5)	0.0362 (18)	0.50
H2A	0.5367	0.8047	0.1481	0.043*	0.50
H2B	0.4341	0.7998	0.1949	0.043*	0.50
C2'	0.4833 (12)	0.7822 (7)	0.1910 (5)	0.0362 (18)	0.50
H2C	0.5208	0.8413	0.1972	0.043*	0.50
H2D	0.4133	0.7876	0.2125	0.043*	0.50
C3	0.5051 (3)	0.5443 (2)	0.3823 (2)	0.0281 (7)	
C4	0.4108 (3)	0.4957 (2)	0.4107 (2)	0.0328 (8)	
H4A	0.4373	0.4402	0.4387	0.039*	
H4B	0.3609	0.4768	0.3650	0.039*	
C5	0.8135 (3)	0.6338 (2)	0.5270 (2)	0.0294 (7)	
C6	0.8464 (3)	0.6291 (3)	0.6136 (2)	0.0370 (8)	
H6A	0.9235	0.6454	0.6239	0.044*	
H6B	0.8384	0.5659	0.6312	0.044*	
C7	0.8616 (3)	0.7876 (2)	0.3679 (2)	0.0270 (7)	
C8	0.9157 (3)	0.8759 (2)	0.3518 (2)	0.0352 (8)	
H8A	0.9299	0.9101	0.4012	0.042*	0.836 (19)
H8B	0.8661	0.9124	0.3156	0.042*	0.836 (19)
H8C	0.8920	0.9223	0.3881	0.042*	0.164 (19)
H8D	0.8904	0.8956	0.2974	0.042*	0.164 (19)
C9	0.8080 (3)	0.4002 (2)	0.3506 (2)	0.0308 (7)	
C10	0.8248 (4)	0.2993 (2)	0.3643 (3)	0.0511 (11)	
H10A	0.8731	0.2754	0.3273	0.061*	0.60 (6)
H10B	0.7549	0.2676	0.3545	0.061*	0.60 (6)
H10C	0.8613	0.2754	0.3200	0.061*	0.40 (6)
H10D	0.7536	0.2688	0.3624	0.061*	0.40 (6)
C11	0.8661 (3)	0.5728 (2)	0.2035 (2)	0.0348 (8)	
C12	0.9326 (13)	0.591 (2)	0.1367 (4)	0.050 (3)	0.50
H12A	1.0062	0.5668	0.1508	0.060*	0.50
H12B	0.9386	0.6571	0.1296	0.060*	0.50
C12'	0.9334 (16)	0.606 (3)	0.1410 (5)	0.050 (3)	0.365 (3)
H12C	0.9942	0.5631	0.1379	0.060*	0.365 (3)
H12D	0.9639	0.6656	0.1562	0.060*	0.365 (3)
C12"	0.9007 (16)	0.576 (4)	0.1220 (7)	0.050 (3)	0.135 (3)
H12E	0.8598	0.6235	0.0906	0.060*	0.135 (3)
H12F	0.8877	0.5172	0.0955	0.060*	0.135 (3)

### Atomic displacement parameters (Å^2^)

**Table:**

	*U*^11^	*U*^22^	*U*^33^	*U*^12^	*U*^13^	*U*^23^
Cr1	0.0183 (2)	0.0182 (2)	0.0442 (3)	0.00070 (18)	0.0065 (2)	0.0001 (2)
Cr2	0.0173 (2)	0.0190 (2)	0.0398 (3)	0.00040 (18)	0.0064 (2)	0.0012 (2)
Cr3	0.0225 (3)	0.0238 (2)	0.0313 (3)	0.00189 (19)	0.0034 (2)	0.0028 (2)
Cl1	0.0588 (17)	0.096 (2)	0.0418 (14)	0.0318 (15)	−0.0030 (12)	−0.0015 (16)
Cl1'	0.091 (2)	0.0727 (19)	0.0409 (14)	0.0348 (16)	−0.0019 (15)	0.0023 (14)
Cl2	0.0289 (4)	0.0431 (5)	0.0559 (6)	−0.0019 (4)	0.0155 (4)	−0.0009 (4)
Cl3	0.0521 (6)	0.0564 (6)	0.0475 (6)	0.0105 (5)	0.0077 (5)	−0.0128 (5)
Cl4	0.0508 (13)	0.0436 (8)	0.101 (3)	−0.0061 (8)	0.0421 (16)	0.0069 (12)
Cl5	0.042 (2)	0.0331 (15)	0.060 (3)	0.0081 (11)	−0.0086 (13)	0.0093 (12)
Cl4'	0.033 (4)	0.041 (4)	0.097 (11)	−0.005 (3)	0.006 (5)	0.017 (6)
Cl5'	0.084 (8)	0.044 (3)	0.093 (4)	0.001 (5)	−0.023 (5)	0.019 (3)
Cl6	0.086 (2)	0.203 (4)	0.0466 (17)	0.021 (3)	0.0250 (15)	0.020 (3)
Cl6'	0.065 (2)	0.111 (3)	0.0340 (17)	−0.020 (2)	0.0111 (15)	0.015 (2)
Cl6"	0.054 (5)	0.058 (5)	0.069 (5)	−0.015 (4)	0.026 (4)	0.000 (4)
O1	0.0346 (13)	0.0363 (13)	0.0418 (15)	0.0098 (11)	0.0000 (11)	0.0077 (11)
O2	0.0364 (15)	0.0282 (13)	0.0600 (19)	0.0087 (11)	−0.0049 (13)	0.0009 (12)
O3	0.0239 (12)	0.0266 (12)	0.0536 (16)	−0.0047 (9)	0.0137 (11)	−0.0050 (11)
O4	0.0295 (13)	0.0355 (13)	0.0396 (15)	−0.0089 (10)	0.0082 (11)	−0.0029 (11)
O5	0.0271 (12)	0.0320 (12)	0.0426 (15)	0.0046 (10)	0.0039 (11)	−0.0025 (11)
O6	0.0234 (12)	0.0420 (14)	0.0406 (15)	0.0044 (10)	0.0068 (11)	0.0003 (11)
O7	0.0251 (12)	0.0233 (11)	0.0525 (16)	−0.0022 (9)	0.0112 (11)	−0.0006 (10)
O8	0.0235 (12)	0.0221 (11)	0.0656 (18)	−0.0021 (9)	0.0055 (12)	0.0051 (11)
O9	0.0259 (12)	0.0196 (11)	0.0616 (17)	−0.0004 (9)	−0.0022 (11)	0.0013 (11)
O10	0.0379 (14)	0.0222 (11)	0.0450 (15)	0.0031 (10)	−0.0050 (12)	0.0023 (10)
O11	0.0291 (13)	0.0504 (16)	0.0457 (17)	0.0016 (11)	0.0128 (12)	−0.0018 (13)
O12	0.0319 (14)	0.0483 (15)	0.0353 (14)	0.0032 (11)	0.0088 (11)	0.0026 (11)
O13	0.0193 (10)	0.0199 (10)	0.0373 (13)	0.0011 (8)	0.0049 (9)	0.0019 (9)
O14	0.0467 (19)	0.085 (3)	0.125 (4)	0.0030 (18)	0.041 (2)	−0.029 (2)
O15	0.0555 (19)	0.0463 (16)	0.081 (2)	−0.0064 (14)	0.0256 (18)	0.0027 (16)
O16	0.078 (2)	0.0499 (19)	0.082 (3)	0.0108 (17)	0.011 (2)	0.0139 (17)
O1w	0.0254 (13)	0.0346 (14)	0.083 (2)	0.0047 (11)	0.0088 (14)	−0.0190 (14)
O2w	0.0197 (11)	0.0342 (13)	0.0490 (16)	0.0008 (10)	0.0070 (11)	0.0052 (12)
O3w	0.0338 (14)	0.0371 (14)	0.0442 (16)	0.0008 (11)	−0.0010 (12)	−0.0075 (12)
O4w	0.0472 (19)	0.065 (2)	0.101 (3)	−0.0105 (16)	0.0167 (19)	−0.037 (2)
O5w	0.142 (5)	0.157 (5)	0.128 (4)	0.046 (4)	0.031 (4)	0.086 (4)
O6w	0.0514 (19)	0.0529 (19)	0.070 (2)	0.0062 (15)	−0.0006 (16)	−0.0111 (16)
O7w	0.181 (9)	0.119 (7)	0.173 (9)	0.006 (7)	0.029 (7)	−0.008 (7)
N1	0.0307 (17)	0.0429 (18)	0.052 (2)	−0.0041 (14)	0.0080 (15)	−0.0089 (15)
C1	0.0211 (16)	0.0286 (17)	0.056 (3)	0.0036 (13)	0.0075 (16)	0.0165 (17)
C2	0.040 (2)	0.026 (4)	0.042 (5)	0.013 (3)	−0.001 (4)	−0.001 (3)
C2'	0.040 (2)	0.026 (4)	0.042 (5)	0.013 (3)	−0.001 (4)	−0.001 (3)
C3	0.0207 (15)	0.0246 (15)	0.038 (2)	0.0001 (12)	0.0006 (14)	0.0055 (14)
C4	0.0259 (17)	0.0333 (17)	0.040 (2)	−0.0053 (14)	0.0072 (15)	−0.0007 (15)
C5	0.0236 (16)	0.0226 (15)	0.043 (2)	−0.0060 (13)	0.0081 (15)	0.0004 (14)
C6	0.0333 (19)	0.0398 (19)	0.038 (2)	0.0028 (15)	0.0062 (16)	−0.0014 (16)
C7	0.0262 (17)	0.0215 (15)	0.0333 (18)	−0.0035 (12)	0.0038 (14)	0.0015 (13)
C8	0.0314 (18)	0.0248 (16)	0.051 (2)	−0.0052 (14)	0.0128 (17)	0.0002 (15)
C9	0.0266 (17)	0.0247 (16)	0.042 (2)	0.0009 (13)	0.0080 (15)	0.0027 (14)
C10	0.047 (2)	0.0234 (18)	0.077 (3)	−0.0011 (16)	−0.019 (2)	0.0082 (18)
C11	0.0323 (19)	0.0287 (17)	0.046 (2)	0.0068 (14)	0.0173 (17)	0.0071 (16)
C12	0.046 (3)	0.054 (9)	0.053 (3)	0.005 (3)	0.025 (3)	0.014 (3)
C12'	0.046 (3)	0.054 (9)	0.053 (3)	0.005 (3)	0.025 (3)	0.014 (3)
C12"	0.046 (3)	0.054 (9)	0.053 (3)	0.005 (3)	0.025 (3)	0.014 (3)

### Geometric parameters (Å, °)

**Table:**

Cr1—O13	1.905 (2)	O1w—H12	0.84 (3)
Cr1—O2	1.957 (3)	O2w—H21	0.84 (3)
Cr1—O3	1.973 (2)	O2w—H22	0.84 (3)
Cr1—O5	1.975 (3)	O3w—H31	0.844 (14)
Cr1—O7	1.994 (2)	O3w—H32	0.84 (3)
Cr1—O1w	2.010 (2)	O4w—H41	0.85 (5)
Cr2—O13	1.901 (2)	O4w—H42	0.83 (4)
Cr2—O11	1.952 (3)	O5w—H51	0.85 (4)
Cr2—O6	1.956 (3)	O5w—H52	0.85 (7)
Cr2—O8	1.967 (2)	O6w—H61	0.83 (4)
Cr2—O9	1.977 (2)	O6w—H62	0.85 (4)
Cr2—O2w	2.041 (2)	O7w—H71	0.84
Cr3—O13	1.906 (2)	O7w—H72	0.84
Cr3—O4	1.964 (2)	C1—C2'	1.522 (7)
Cr3—O10	1.975 (2)	C1—C2	1.525 (7)
Cr3—O1	1.978 (2)	C2—H2A	0.9900
Cr3—O12	1.984 (2)	C2—H2B	0.9900
Cr3—O3w	2.013 (3)	C2'—H2C	0.9900
Cl1—C2	1.741 (8)	C2'—H2D	0.9900
Cl1'—C2'	1.738 (8)	C3—C4	1.507 (4)
Cl2—C4	1.772 (3)	C4—H4A	0.9900
Cl3—C6	1.763 (4)	C4—H4B	0.9900
Cl4—C8	1.762 (4)	C5—C6	1.508 (5)
Cl4'—C8	1.745 (8)	C6—H6A	0.9900
Cl5'—C10	1.770 (8)	C6—H6B	0.9900
Cl6—C12	1.742 (11)	C7—C8	1.507 (4)
Cl6'—C12'	1.744 (12)	C8—H8A	0.9900
Cl6"—C12"	1.741 (13)	C8—H8B	0.9900
O1—C1	1.252 (4)	C8—H8C	0.9900
O2—C1	1.236 (5)	C8—H8D	0.9900
O3—C3	1.247 (4)	C9—C10	1.518 (4)
O4—C3	1.254 (4)	C10—H10A	0.9900
O5—C5	1.247 (4)	C10—H10B	0.9900
O6—C5	1.255 (4)	C10—H10C	0.9900
O7—C7	1.258 (4)	C10—H10D	0.9900
O8—C7	1.242 (4)	C11—C12	1.517 (7)
O9—C9	1.246 (4)	C11—C12'	1.518 (8)
O10—C9	1.244 (4)	C11—C12"	1.518 (10)
O11—C11	1.243 (5)	C12—H12A	0.9900
O12—C11	1.249 (4)	C12—H12B	0.9900
O14—N1	1.233 (4)	C12'—H12C	0.9900
O15—N1	1.230 (4)	C12'—H12D	0.9900
O16—N1	1.234 (4)	C12"—H12E	0.9900
O1w—H11	0.84 (4)	C12"—H12F	0.9900
			
O13—Cr1—O2	95.69 (10)	C1—C2—Cl1	121.0 (6)
O13—Cr1—O3	93.62 (9)	C1—C2—H2A	107.1
O2—Cr1—O3	90.71 (11)	Cl1—C2—H2A	107.1
O13—Cr1—O5	96.24 (10)	C1—C2—H2B	107.1
O2—Cr1—O5	168.02 (10)	Cl1—C2—H2B	107.1
O3—Cr1—O5	89.64 (11)	H2A—C2—H2B	106.8
O13—Cr1—O7	94.29 (9)	C1—C2'—Cl1'	108.6 (5)
O2—Cr1—O7	86.43 (11)	C1—C2'—H2C	110.0
O3—Cr1—O7	171.82 (9)	Cl1'—C2'—H2C	110.0
O5—Cr1—O7	91.58 (11)	C1—C2'—H2D	110.0
O13—Cr1—O1w	177.12 (12)	Cl1'—C2'—H2D	110.0
O2—Cr1—O1w	81.71 (13)	H2C—C2'—H2D	108.3
O3—Cr1—O1w	85.20 (10)	O3—C3—O4	127.1 (3)
O5—Cr1—O1w	86.39 (12)	O3—C3—C4	119.6 (3)
O7—Cr1—O1w	86.81 (10)	O4—C3—C4	113.2 (3)
O13—Cr2—O11	94.36 (11)	C3—C4—Cl2	114.1 (2)
O13—Cr2—O6	96.91 (10)	C3—C4—H4A	108.7
O11—Cr2—O6	168.71 (11)	Cl2—C4—H4A	108.7
O13—Cr2—O8	92.98 (9)	C3—C4—H4B	108.7
O11—Cr2—O8	87.27 (11)	Cl2—C4—H4B	108.7
O6—Cr2—O8	91.41 (11)	H4A—C4—H4B	107.6
O13—Cr2—O9	95.36 (9)	O5—C5—O6	126.6 (3)
O11—Cr2—O9	93.14 (11)	O5—C5—C6	120.1 (3)
O6—Cr2—O9	86.55 (11)	O6—C5—C6	113.3 (3)
O8—Cr2—O9	171.60 (10)	C5—C6—Cl3	114.4 (3)
O13—Cr2—O2w	177.32 (10)	C5—C6—H6A	108.7
O11—Cr2—O2w	83.58 (11)	Cl3—C6—H6A	108.7
O6—Cr2—O2w	85.14 (10)	C5—C6—H6B	108.7
O8—Cr2—O2w	85.22 (10)	Cl3—C6—H6B	108.7
O9—Cr2—O2w	86.48 (10)	H6A—C6—H6B	107.6
O13—Cr3—O4	93.39 (10)	O8—C7—O7	126.9 (3)
O13—Cr3—O10	95.01 (10)	O8—C7—C8	118.4 (3)
O4—Cr3—O10	88.96 (11)	O7—C7—C8	114.7 (3)
O13—Cr3—O1	96.30 (10)	C7—C8—Cl4'	114.3 (4)
O4—Cr3—O1	91.39 (11)	C7—C8—Cl4	113.0 (2)
O10—Cr3—O1	168.65 (11)	C7—C8—H8A	109.0
O13—Cr3—O12	94.62 (10)	Cl4—C8—H8A	109.0
O4—Cr3—O12	171.88 (11)	C7—C8—H8B	109.0
O10—Cr3—O12	91.67 (11)	Cl4—C8—H8B	109.0
O1—Cr3—O12	86.40 (10)	H8A—C8—H8B	107.8
O13—Cr3—O3w	178.83 (10)	C7—C8—H8C	108.0
O4—Cr3—O3w	85.83 (11)	C7—C8—H8D	108.8
O10—Cr3—O3w	85.86 (11)	H8C—C8—H8D	107.8
O1—Cr3—O3w	82.86 (11)	O10—C9—O9	127.4 (3)
O12—Cr3—O3w	86.14 (11)	O10—C9—C10	113.8 (3)
C1—O1—Cr3	130.9 (3)	O9—C9—C10	118.8 (3)
C1—O2—Cr1	134.1 (2)	C9—C10—Cl5'	110.9 (8)
C3—O3—Cr1	131.5 (2)	C9—C10—H10A	109.4
C3—O4—Cr3	132.8 (2)	Cl5'—C10—H10A	109.5
C5—O5—Cr1	133.3 (2)	C9—C10—H10B	109.4
C5—O6—Cr2	131.1 (2)	Cl5'—C10—H10B	109.4
C7—O7—Cr1	129.0 (2)	H10A—C10—H10B	108.0
C7—O8—Cr2	134.6 (2)	C9—C10—H10C	107.1
C9—O9—Cr2	129.6 (2)	C9—C10—H10D	109.3
C9—O10—Cr3	133.6 (2)	H10C—C10—H10D	107.3
C11—O11—Cr2	134.1 (2)	O11—C11—O12	126.9 (3)
C11—O12—Cr3	129.5 (3)	O11—C11—C12	112.6 (5)
Cr2—O13—Cr1	119.84 (12)	O12—C11—C12	120.5 (5)
Cr2—O13—Cr3	119.75 (11)	O11—C11—C12'	109.3 (5)
Cr1—O13—Cr3	120.41 (11)	O12—C11—C12'	123.4 (6)
Cr1—O1w—H11	130 (3)	O11—C11—C12"	130.6 (7)
Cr1—O1w—H12	120 (3)	O12—C11—C12"	102.2 (7)
H11—O1w—H12	110 (4)	C11—C12—Cl6	113.9 (6)
Cr2—O2w—H21	124 (3)	C11—C12—H12A	108.8
Cr2—O2w—H22	118 (3)	Cl6—C12—H12A	108.8
H21—O2w—H22	108 (4)	C11—C12—H12B	108.8
Cr3—O3w—H31	113 (3)	Cl6—C12—H12B	108.8
Cr3—O3w—H32	128 (3)	H12A—C12—H12B	107.7
H31—O3w—H32	110 (4)	C11—C12'—Cl6'	113.2 (7)
H41—O4w—H42	108 (5)	C11—C12'—H12C	108.9
H51—O5w—H52	108 (6)	Cl6'—C12'—H12C	108.9
H61—O6w—H62	110 (4)	C11—C12'—H12D	108.9
H71—O7w—H72	110	Cl6'—C12'—H12D	108.9
O15—N1—O14	118.7 (4)	H12C—C12'—H12D	107.8
O15—N1—O16	120.7 (3)	C11—C12"—Cl6"	106.6 (8)
O14—N1—O16	120.5 (4)	C11—C12"—H12E	110.4
O2—C1—O1	126.9 (3)	Cl6"—C12"—H12E	110.4
O2—C1—C2'	104.2 (4)	C11—C12"—H12F	110.4
O1—C1—C2'	128.9 (5)	Cl6"—C12"—H12F	110.4
O2—C1—C2	121.9 (4)	H12E—C12"—H12F	108.6
O1—C1—C2	111.2 (5)		
			
O13—Cr3—O1—C1	−23.1 (3)	O5—Cr1—O13—Cr3	139.83 (13)
O4—Cr3—O1—C1	70.4 (3)	O7—Cr1—O13—Cr3	−128.11 (13)
O10—Cr3—O1—C1	162.1 (5)	O4—Cr3—O13—Cr2	131.56 (13)
O12—Cr3—O1—C1	−117.4 (3)	O10—Cr3—O13—Cr2	42.31 (14)
O3w—Cr3—O1—C1	156.1 (3)	O1—Cr3—O13—Cr2	−136.66 (13)
O13—Cr1—O2—C1	18.0 (3)	O12—Cr3—O13—Cr2	−49.78 (14)
O3—Cr1—O2—C1	−75.8 (3)	O4—Cr3—O13—Cr1	−48.53 (14)
O5—Cr1—O2—C1	−167.4 (4)	O10—Cr3—O13—Cr1	−137.78 (13)
O7—Cr1—O2—C1	111.9 (3)	O1—Cr3—O13—Cr1	43.25 (14)
O1w—Cr1—O2—C1	−160.8 (4)	O12—Cr3—O13—Cr1	130.13 (13)
O13—Cr1—O3—C3	−25.9 (3)	Cr1—O2—C1—O1	1.1 (6)
O2—Cr1—O3—C3	69.9 (3)	Cr1—O2—C1—C2'	−179.6 (7)
O5—Cr1—O3—C3	−122.1 (3)	Cr1—O2—C1—C2	−177.3 (8)
O1w—Cr1—O3—C3	151.5 (3)	Cr3—O1—C1—O2	2.1 (5)
O13—Cr3—O4—C3	22.5 (3)	Cr3—O1—C1—C2'	−177.0 (9)
O10—Cr3—O4—C3	117.5 (3)	Cr3—O1—C1—C2	−179.3 (7)
O1—Cr3—O4—C3	−73.9 (3)	O2—C1—C2—Cl1	−156.9 (7)
O3w—Cr3—O4—C3	−156.6 (3)	O1—C1—C2—Cl1	24.5 (13)
O13—Cr1—O5—C5	17.5 (3)	C2'—C1—C2—Cl1	−150 (5)
O2—Cr1—O5—C5	−157.2 (5)	O2—C1—C2'—Cl1'	171.1 (7)
O3—Cr1—O5—C5	111.1 (3)	O1—C1—C2'—Cl1'	−9.6 (14)
O7—Cr1—O5—C5	−77.0 (3)	C2—C1—C2'—Cl1'	−2(3)
O1w—Cr1—O5—C5	−163.7 (3)	Cr1—O3—C3—O4	3.7 (5)
O13—Cr2—O6—C5	−27.7 (3)	Cr1—O3—C3—C4	−178.5 (2)
O11—Cr2—O6—C5	148.5 (5)	Cr3—O4—C3—O3	−1.6 (6)
O8—Cr2—O6—C5	65.4 (3)	Cr3—O4—C3—C4	−179.5 (2)
O9—Cr2—O6—C5	−122.7 (3)	O3—C3—C4—Cl2	16.3 (4)
O2w—Cr2—O6—C5	150.5 (3)	O4—C3—C4—Cl2	−165.7 (3)
O13—Cr1—O7—C7	−25.0 (3)	Cr1—O5—C5—O6	−1.5 (5)
O2—Cr1—O7—C7	−120.4 (3)	Cr1—O5—C5—C6	179.5 (2)
O5—Cr1—O7—C7	71.4 (3)	Cr2—O6—C5—O5	7.9 (5)
O1w—Cr1—O7—C7	157.7 (3)	Cr2—O6—C5—C6	−173.1 (2)
O13—Cr2—O8—C7	14.9 (4)	O5—C5—C6—Cl3	−10.9 (4)
O11—Cr2—O8—C7	109.2 (4)	O6—C5—C6—Cl3	170.0 (2)
O6—Cr2—O8—C7	−82.1 (4)	Cr2—O8—C7—O7	10.5 (6)
O2w—Cr2—O8—C7	−167.1 (4)	Cr2—O8—C7—C8	−170.1 (3)
O13—Cr2—O9—C9	30.3 (3)	Cr1—O7—C7—O8	−3.9 (6)
O11—Cr2—O9—C9	−64.4 (3)	Cr1—O7—C7—C8	176.8 (2)
O6—Cr2—O9—C9	126.9 (3)	O8—C7—C8—Cl4'	−8.4 (14)
O2w—Cr2—O9—C9	−147.8 (3)	O7—C7—C8—Cl4'	171.0 (14)
O13—Cr3—O10—C9	−11.4 (3)	O8—C7—C8—Cl4	17.8 (6)
O4—Cr3—O10—C9	−104.7 (3)	O7—C7—C8—Cl4	−162.8 (4)
O1—Cr3—O10—C9	163.4 (5)	Cr3—O10—C9—O9	−7.9 (6)
O12—Cr3—O10—C9	83.4 (3)	Cr3—O10—C9—C10	174.4 (3)
O3w—Cr3—O10—C9	169.4 (3)	Cr2—O9—C9—O10	−4.0 (5)
O13—Cr2—O11—C11	−10.5 (3)	Cr2—O9—C9—C10	173.5 (3)
O6—Cr2—O11—C11	173.2 (5)	O10—C9—C10—Cl5'	−149.7 (16)
O8—Cr2—O11—C11	−103.3 (3)	O9—C9—C10—Cl5'	32.4 (16)
O9—Cr2—O11—C11	85.1 (3)	Cr2—O11—C11—O12	−14.5 (6)
O2w—Cr2—O11—C11	171.2 (3)	Cr2—O11—C11—C12	166.4 (14)
O13—Cr3—O12—C11	25.2 (3)	Cr2—O11—C11—C12'	158 (2)
O10—Cr3—O12—C11	−70.0 (3)	Cr2—O11—C11—C12"	173 (3)
O1—Cr3—O12—C11	121.2 (3)	Cr3—O12—C11—O11	4.9 (5)
O3w—Cr3—O12—C11	−155.7 (3)	Cr3—O12—C11—C12	−176.1 (15)
O11—Cr2—O13—Cr1	−135.27 (13)	Cr3—O12—C11—C12'	−166 (2)
O6—Cr2—O13—Cr1	44.00 (14)	Cr3—O12—C11—C12"	179 (2)
O8—Cr2—O13—Cr1	−47.79 (14)	O11—C11—C12—Cl6	156.8 (13)
O9—Cr2—O13—Cr1	131.16 (13)	O12—C11—C12—Cl6	−22 (2)
O11—Cr2—O13—Cr3	44.64 (14)	C12'—C11—C12—Cl6	−134 (12)
O6—Cr2—O13—Cr3	−136.09 (13)	C12"—C11—C12—Cl6	−8(7)
O8—Cr2—O13—Cr3	132.12 (14)	O11—C11—C12'—Cl6'	−172 (2)
O9—Cr2—O13—Cr3	−48.93 (14)	O12—C11—C12'—Cl6'	1(4)
O2—Cr1—O13—Cr2	138.63 (13)	C12—C11—C12'—Cl6'	74 (8)
O3—Cr1—O13—Cr2	−130.29 (13)	C12"—C11—C12'—Cl6'	36 (5)
O5—Cr1—O13—Cr2	−40.26 (14)	O11—C11—C12"—Cl6"	−5(4)
O7—Cr1—O13—Cr2	51.80 (14)	O12—C11—C12"—Cl6"	−179 (2)
O2—Cr1—O13—Cr3	−41.28 (14)	C12—C11—C12"—Cl6"	14 (6)
O3—Cr1—O13—Cr3	49.80 (14)	C12'—C11—C12"—Cl6"	31 (5)

### Hydrogen-bond geometry (Å, °)

**Table:**

*D*—H···*A*	*D*—H	H···*A*	*D*···*A*	*D*—H···*A*
O1w—H11···O5w^i^	0.84 (4)	1.83 (2)	2.616 (6)	157 (5)
O1w—H12···O6w^ii^	0.84 (3)	1.99 (3)	2.816 (4)	169 (5)
O2w—H21···O15^iii^	0.84 (3)	1.90 (3)	2.732 (4)	170 (4)
O2w—H22···CL5'^iv^	0.84 (3)	2.75 (3)	3.295 (6)	124 (6)
O3w—H31···O4w	0.844 (14)	1.84 (2)	2.669 (4)	168 (5)
O3w—H32···O6w	0.84 (3)	1.88 (3)	2.711 (4)	171 (4)
O4w—H41···O7^v^	0.85 (5)	2.20 (5)	3.046 (4)	175 (5)
O4w—H42···O16^ii^	0.83 (4)	2.27 (4)	2.976 (5)	142 (6)
O5w—H51···O7w	0.85 (4)	2.06 (7)	2.79 (1)	144 (11)
O5w—H52···O12	0.85 (7)	2.47 (7)	3.27 (1)	156 (10)
O6w—H61···O14	0.83 (4)	2.31 (2)	3.093 (6)	158 (5)
O6w—H62···O7w	0.85 (4)	2.15 (3)	2.86 (1)	141 (4)

Symmetry codes: (i) *x*, −*y*+3/2, *z*+1/2; (ii) −*x*+1, *y*+1/2, −*z*+1/2; (iii) −*x*+2, *y*+1/2, −*z*+1/2; (iv) −*x*+2, −*y*+1, −*z*+1; (v) −*x*+1, *y*−1/2, −*z*+1/2.
